# Positive regulation of Vav1 by Themis controls CD4 T cell pathogenicity in a mouse model of central nervous system inflammation

**DOI:** 10.1007/s00018-024-05203-5

**Published:** 2024-04-02

**Authors:** Remi Marrocco, Isabelle Bernard, Emeline Joulia, Rebecca Barascud, Anne S. Dejean, Renaud Lesourne, Abdelhadi Saoudi

**Affiliations:** 1 Institut Toulousain des Maladies Infectieuses Et Inflammatoires (Infinity), Université de Toulouse, Centre National de la Recherche Scientifique (CNRS), Institut National de la Santé et de la Recherche Médicale (Inserm), INSERM U1291, Université Paul Sabatier (UPS), CHU Purpan, BP 3028, 31024 Toulouse Cedex 3, France; 2 Present Address: Division of Immune Regulation, La Jolla Institute for Immunology, La Jolla, CA USA; 3 Present Address: Molecular and Cell Biology Laboratory, Salk Institute for Biological Studies, La Jolla, CA 92037 USA

**Keywords:** TCR, Epistasis, Signaling, Susceptibility gene, Autoimmunity

## Abstract

The susceptibility to autoimmune diseases is conditioned by the association of modest genetic alterations which altogether weaken self-tolerance. The mechanism whereby these genetic interactions modulate T-cell pathogenicity remains largely uncovered. Here, we investigated the epistatic interaction of two interacting proteins involved in T Cell Receptor signaling and which were previously associated with the development of Multiple Sclerosis. To this aim, we used mice expressing an hypomorphic variant of Vav1 (Vav1^R63W^), combined with a T cell-conditional deletion of Themis. We show that the combined mutations in Vav1 and Themis induce a strong attenuation of the severity of Experimental Autoimmune Encephalomyelitis (EAE), contrasting with the moderate effect of the single mutation in each of those two proteins. This genotype-dependent gradual decrease of EAE severity correlates with decreased quantity of phosphorylated Vav1 in CD4 T cells, establishing that Themis promotes the development of encephalitogenic Tconv response by enhancing Vav1 activity. We also show that the cooperative effect of Themis and Vav1 on EAE severity is independent of regulatory T cells and unrelated to the impact of Themis on thymic selection. Rather, it results from decreased production of pro-inflammatory cytokines (IFN-γ, IL-17, TNF and GM-CSF) and reduced T cell infiltration in the CNS. Together, our results provide a rationale to study combination of related genes, in addition to single gene association, to better understand the genetic bases of human diseases.

**Supplementary Information:**

The online version contains supplementary material available at 10.1007/s00018-024-05203-5.

## Introduction

Autoimmune disorders are a complex and diverse group of diseases that are caused by the rupture of self-tolerance. The etiology of autoimmunity is multifactorial, with environmental triggers and genetically-determined risk factors. Genome-wide association studies (GWAS) have identified hundreds of genes associated with human diseases, notably for Multiple Sclerosis (MS), but the assessment of the actual pathophysiological link and underlying mechanisms are still mostly unknown. In addition, only less than 40% of risk heritability is attributable to the common genetic variants identified by GWAS [1]. Other potential contributors to heritability include gene-environment interactions and gene–gene interactions. Indeed, several studies showed that disease-causing polymorphisms show wide phenotypic variability, even within families, emphasizing that mutation outcome is dependent on the genetic background, as well as on environmental factors [2]. Next-generation sequencing (NGS) analysis has revealed that a proportion of individuals might carry mutations in one or more genes controlling susceptibility to inflammatory diseases [3]. These observations highlight the need to study combination of related genes, rather than single associations, to better decipher the physiopathology and etiology of human immune-mediated diseases.

We previously identified Themis (THymocyte-Expressed Molecule Important for Selection) as a T-cell signaling protein essential for the full development of CD4^+^ and CD8^+^ thymocytes [4, 5]. Themis is recruited to TCR signaling complexes via the cytosolic adapter Grb2, which binds to the transmembrane adaptor LAT following TCR engagement [6]. Themis interacts constitutively with the tyrosine phosphatase SHP-1 (Src homology region 2 domain-containing phosphatase-1) [7–9], which exerts well-characterized inhibitory functions on TCR signaling [10, 11]. It was suggested that Themis negatively regulates the TCR signals by recruiting SHP-1 close to TCR-signaling clusters [9, 12]. However, recent studies established rather that Themis enhances TCR signals by repressing the catalytic activity of SHP-1 [8, 13–16]. Genetic studies suggest an association between genetic variants on *THEMIS* and the risk of developing MS [1, 17, 18]. In agreement, we recently showed in EAE mouse models that Themis enhances the encephalitogenicity of CD4 T cells by favoring their migration into the central nervous system and by enhancing the magnitude of Th1 responses [19].

Functional cooperation between the Themis and Vav1 has been suggested by several studies. Indeed, the disruption of the *Themis* gene in BN but not LEW rats leads to the spontaneous development of inflammatory bowel disease (IBD) and genetic dissection revealed that this phenotype is only observed in rats that combine Themis deficiency with a locus of 117 Kb that contains the Vav1^R63W^ variant [5, 20]. Vav1 is a key signal transducer downstream of the TCR and is mandatory for the development and activation of T cells [21, 22]. Following TCR engagement, Vav1 becomes rapidly tyrosine phosphorylated, which allows Vav1 to promote GDP-GTP exchange on the Rho, Rac1 and Cdc42 small GTPases [23, 24]. In addition to this GEF activity, the CH, SH2 and SH3 domains of Vav1 exert molecular adapter functions and contribute to the assembly of LAT-Grb2-Themis-Gads-PLCγ1-SLP76 signaling micro-clusters which are induced after TCR stimulation [22, 25, 26]. It has been shown that many critical events involved in T cell activation are mediated by either the GEF or the scaffolding activities of Vav1 [27]. The Vav1^R63W^ variant leads to an impaired stability of the Vav1, thereby decreasing its adaptor function without impacting its GEF activity, resulting in attenuation of TCR-mediated signaling events [28, 29]. Additionally, we previously showed that the expression of the Vav1^R63W^ variant in Themis-sufficient mice attenuates T-cell encephalitogenic responses in the EAE model [29]. Finally, several studies revealed a close relation between these two signaling molecules. Indeed, analysis of the Themis and Vav1 interactome by mass spectrometry revealed that these molecules are privileged binding partners in T cells [7, 25]. Moreover, it has been shown that Themis positively regulates Vav1 activity in thymocytes [7]. Finally, Vav1 can be dephosphorylated by SHP-1, which decreases its activity [30, 31]. Since Themis stimulates the catalytic activity of Vav1 and since the R63W mutation reduces its scaffold function, we hypothesized that the combination of both mutations may have a cooperative effect on Vav1 activity which may ultimately affect the magnitude of T-cell pathogenic responses and the development of CNS inflammation.

To this end, we crossed conditional knock-out (cKO) mice lacking Themis in peripheral T cells, but not in thymocytes [19], with Vav1^R63W^ knock-in (KI) mice [29]. We next analyzed the effect of single, *vs.* combined mutations, on the development of EAE. Here, we show that EAE severity is strongly reduced in KI-cKO mice as compared to mice with single mutation (KI or cKO) and that this is correlated with the amount of Vav1 phosphorylation upon TCR stimulation. The effect of the double mutation on EAE severity is unrelated to the impact of Themis on thymic selection, but rather results from reduced T cell infiltration in the CNS. We also provide evidence that this reduced infiltration of inflammatory cells into the CNS in KI-cKO mice is not the consequence of their impaired migratory capacity, but rather of their incapacity to produce inflammatory cytokines, key factors that disrupt the blood–brain-barrier (BBB) integrity. Together, our study reveals an epistatic link between Themis and Vav1 that influences the activity of Vav1 and the development of EAE by controlling the effector function of encephalitogenic CD4 conventional T cells (Tconv).

## Materials and methods

### Mice

The Vav1^R63W^ knock-in (KI) mice (international strain designation C57BL/6-Vav1tm2Mal) were generated in collaboration with Dr B. Malissen [29]. Themis-T^−/−^ conditional knock-out mice (cKO) were generated in collaboration with Dr P. Love [19]. The Vav1^R63W^ mice were crossed with Themis-T^−/−^ mice to generate Vav1^R63W^Themis-T^−/−^ mice (KI-cKO). Themis^loxp/loxp^ mice were also bred with CD4CreERT2 mice, which contain tamoxifen-inducible Cre under the control of the *Cd4* promoter (https://www.jax.org/strain/022356). RAG2-KO Rag2^−/−^ mice were provided by the INSERM animal facility (US-006). All experiments were conducted with sex- and age-matched mice between 8 and 12 weeks old housed under specific pathogen-free conditions at the INSERM animal facility (US-006; accreditation number A-31 55,508 delivered by the French Ministry of Agriculture to perform experiments on live mice). All experimental protocols were approved by a Ministry-approved ethics committee (CEEA-122) and follow the French and European regulations on the care and protection of Laboratory Animals (EC Directive 2010/63).

### Experimental auto-immune encephalomyelitis induction and clinical investigation

The MOG_35–55_ (MEVGWYRSPFSRVVHLYRNGK) peptide was purchased from COVALAB with a purity grade > 95%. 8- to 12-week-old mice were immunized subcutaneously at the base of the tail with 200 µL of an emulsion of 0.5 mg/mL of MOG_35–55_ peptide in CFA containing 0.5 mg/mL of Mycobacterium tuberculosis (Strain H37, Difco Franklin Lakes NJ). Mice were injected intravenously with 200 ng of pertussis toxin (List Biological Laboratories, Campbell, CA.) at days 0 and 2 post-immunization. Clinical scores were recorded daily as follow: 0, no signs of disease; 1, loss of tone in the tail; 2, hind limb paresis; 3, hind limb paralysis; 4, tetraplegia; 5, moribund.

For adoptive transfer experiments, a total of 13.10^6^ CD4 T cells were injected i.v. in RAG2-KO mice which were immunized 5 days later day with the protocol mentioned above.

For Treg depletion experiments, mice were treated with 500 µg of PC61 antibody i.p. and immunized 7 days later with the protocol mentioned above. For co-transfer experiment, CD25^−^ CD4 T cells were enriched from LN and spleen of the indicated mice using the Dynabeads negative selection kit (Invitrogen) according to the manufacturer’s instructions with the addition of a PC61 antibody. A total of 40.10^6^ CD25^−^ CD4 T cells were injected i.v. in RAG2-KO mice which were immunized the same day with the protocol mentioned above. For EAE induction in CD4creERT2 mice, each mouse received 5 mg of tamoxifen by oral gavage for 3 days, and were then immunized 12 to 14 days later. Tamoxifen was dissolved in 10% ethanol and 90% Miglyol-812 (Caelo). The emulsion concentration was increased to 1 mg/mL of MOG_35–55_ peptide and 2 mg/mL of Mycobacterium tuberculosis, and PTX quantity remained 200 ng per injection at days 0 and 2 post-immunization.

### Antibodies for flow cytometry

The monoclonal antibodies (mAbs) used for flow cytometry were as follows: anti-TCRαβ (H57-597), anti-CD4 (RM4-5), anti-CD8α (53–6.7), anti-CD44 (IM7), anti-CD25 (PC61), anti-CD62L (MEL-14), anti-CD45 (30-F11), anti-CD45.1 (A20), anti-CD45.2 (104), anti-CD69 (H1.2F3), anti-CCR2 (SA203G11), anti-CCR5 (HM-CCR5), anti-CCR6 (29-2L17), anti-CXCR3 (CXCR3-173), anti-CD11a (M17-4), anti-CD49d (R1-2), anti-KLRG1 (2F1/KLRG1), anti-NK1.1 (PK136), anti-CD11b (M170), anti-CD11c (HL3), anti-Ly6C (HK1.4), anti-Ly6G (1A8), anti-CD80 (16-10A1), anti-MHC-II (M5/114.5.2), anti-Foxp3 (FJK-165), anti-Ki67 (B56) anti-IL-17A (TC11-18H10.1), anti-TNF (MP6-XT-22), anti-GM-CSF (MP1-22E9), anti-IFN-γ (XMG1.2) and anti-T-bet (4B10). The fluorescently conjugated antibodies were purchased from e-Biosciences, BD Biosciences, and Biolegend.

### Isolation and functional characterization of mononuclear cells

Mice were anesthetized with Ketamine and xylazine and perfused with cold PBS. Brain and spinal cord were collected separately, homogenized and digested with collagenase D (2.5 mg/ml, Roche Diagnostics), Dnase I (10 μg/ml) and TLCK (1 μg/ml, Roche, Basel, Switzerland) for 30 to 60 min at 37 ºC. Digestion was stopped by adding cold media and by putting the tubes on ice. Cells were then filtered through a 70 µm cell strainer. After sedimentation, supernatant was collected, resuspended in 30% Percoll and centrifuged for 20 min at 1590 g to allow separation of cells and myelin fat. The pellet was then washed and resuspended in culture medium. Isolated cells were counted using a cell counter (Beckman Coulter) and then directly stained for FACS analysis or restimulated overnight with MOG_35–55_ peptide (100 µg/mL) at 37 °C, in a humidified 5% CO_2_ atmosphere to assess cytokine production using intracytoplasmic staining. Similarly, cells from the dLN cells were either stained directly, stimulated with different concentrations of MOG_35–55_ (0, 10 and 100 μg/mL) for 24 or 48 h to investigate cytokine expression, or stained with CTV (cell trace violet) and then stimulated with MOG_35–55_ (0, 10 and 100 μg/mL) to analyze Tconv proliferation. For intracellular cytokine staining, cells were treated with monensin (GolgiStop 1 μg/ml, BD Biosciences) for the last 4 h of the MOG restimulation. After staining of surface markers, cells were fixed and permeabilized with Foxp3/transcription factor staining buffer set (eBioscience ref. 00-5523-00) according to the manufacturer’s instructions. Cells were then incubated with antibodies recognizing cytokines and transcription factors for 20 min and washed with Perm/Wash buffer before analysis. For tetramer staining, cells from the brain, spinal cord and LNs were incubated for 1.5 h at room temperature with I-Ab MOG_35–55_ tetramer (PE or BV421-coupled, NIH Tetramer core facility), and then stained for surface markers by flow cytometry analysis. Data were collected on an LSR-Fortessa, a FortessaX20 or a Symphony A5 flow cytometers (BD Biosciences) and analyzed with FlowJo software (TreeStar).

The supernatants were assayed for cytokines production by CBA (IL-17, TNF, GM-CSF, IL-6, IL-1b, IL-10, IL-27) (Biolegend, LEGENDplex) or by ELISA for IFN-γ. 96 well plates were coated for 2 h at 37 °C with anti-IFN-γ in carbonate buffer 0.05 M pH 9.6. Culture supernatants or standards were incubated 2 h at 37 °C. The plates were then incubated for 90 min with a secondary biotinylated antibody specific for IFN-γ, followed by 40 min incubation with streptavidin-phosphatase alkaline at 37 °C. Finally, plates were revealed by phosphatase alkaline substrate and absorbance was measured at 405/540 nm.

### Western blotting

Purified total CD4 T cells (8 million) were stimulated for the indicated time in RPMI with pre-formed complex of biotin anti-CD3 (30 µg/mL) and biotin anti-CD4 antibodies (30 µg/mL), linked with streptavidin (15 µg/mL). The stimulation was stopped by adding cold media and the cells were then immediately centrifuged, resuspended in ice-cold lysis buffer [10 mM tris–HCl (pH 7.4), 150 mM NaCl, 1% Triton, 2 mM Na_3_VO_4_, 5 mM NaF, 1 mM EDTA, and protease inhibitor cocktail tablet (Roche)] and incubated for 15–30 min on ice. Total lysates were cleared by centrifugation at 13,000 g for 15 min at 4 °C, harvested and SDS containing loading buffer (NuPAGE 4X) was added to the lysate. Samples were heated for 10 min at 70 °C to allow denaturation of the proteins, resolved on SDS-PAGE gels (Invitrogen Midi-gel) and transferred to PVDF membranes (Biorad Trans-Blot turbo 0.2 µm PVDF). Membranes were blocked in 5% bovine serum albumin or non-fat dry milk in TBS 0.1% Tween-20 for 1 h at room temperature before overnight incubation at 4 °C with the anti-pVav1-Y174 (ab76225) or the anti-GAPDH (5174 s). The membranes were then washed and incubated with horseradish peroxidase-conjugated secondary antibody for 1 h at room temperature. After washing, the membranes were incubated with enhanced chemiluminescence ECL substrate (Amersham), and luminescence was captured with a Bio-Rad XRS + imager. Images were analyzed, and band intensities were quantitated with Bio-Rad ImageLab software. The relative abundance of phospho-Vav1 was determined as a ratio of the intensity of p-Vav1 to that of GAPDH. After revelation of phospho-Vav1, membranes were washed in H_2_O, stripped with Restore buffer (Thermofisher Scienctific) for 15 min, washed and re-blocked for 1 h. Membrane were then incubated overnight with the primary antibodies directed against total Vav1 using anti-Vav1 (SAB4503067), and followed the protocol as described above. Antibodies were purchased from Abcam, Merck and Cell Signaling Technology.

### Assessment of Themis deletion following tamoxifen treatment

Themis^flox/flox^ mice expressing or not the CD4-creERT2 have been treated with tamoxifen as previously described. At different time after treatment, spleen and lymph nodes were harvested, homogenized, and lysed in ACK buffer, before staining the cells for FACS cell sorting for TCR, CD4, CD8 and CD19. B cells were sorted as CD19^+^TCR^−^CD4^−^CD8^−^; CD8 T cells as CD19^−^TCR^+^CD4^−^CD8^+^; and CD4 T cells as CD19^−^TCR^+^CD4^+^CD8^−^. All cells were sorted using FACSAria SORP (BD Biosciences).

DNA was extracted from ~ 10^6^ cells of each the 3 populations sorted (B cells, CD8 T cells, CD4 T cells) using Quiagen blood and sample extraction kit, according to the manufacturer’s instructions. DNA was then quantified with a nanodrop, aliquoted, and stored at − 20 °C. DNA amount was normalized by diluting it in ultrapure RNase-free DNase water. Primers have been designed with BLAST on NCBI and tested in silico by BLAT on UCSC. Used primers were as follow: Forward primer: ATCCCATAGCTCCACCCAAC; Reverse primer: AGAACCTGGGAACGCTAAGTC. Due to the important size of the amplicon with primers PF2 and PR2 (between 1.6 and 3.4kpb), we used the Phusion High-Fidelity DNA Polymerase (ThermoScientific).

### Data analysis

Data are presented as means ± SEM. The GraphPad Prism statistical package was used for statistical analyses (GraphPad Software, Inc.). Results were compared by one-way ANOVA with Tukey correction, and repeated measures by two-way A NOVA with Bonferroni post-test analysis. Mann–Whitney statistical analysis was used when only 2 groups were compared. Results were considered statistically significant when *P* < 0.05 and are indicated in the Figures as follows: *P < 0.05; **P < 0.01; ***P < 0.001; ****P < 0.0001.

## Results

### Impact of combined mutations in Themis and Vav1 genes on T cell homeostasis

Mice bearing a germline deficiency for Themis exhibit a strong lymphopenia, which is caused mainly by a defect of T cell development in the thymus [4]. Since such lymphopenia could modulate the susceptibility to develop autoimmunity, we decided to use mice harboring a conditional disruption of *Themis* in peripheral T cells, but not in thymocytes (_late_CD2-Cre Themis^flox/flox^; named hereafter cKO mice) [19] (Fig. S1A). To study the epistatic interaction of Themis and Vav1 on peripheral T cell responses in the context of CNS inflammation, cKO mice were crossed with Vav1^R63W^ knock-in (KI) mice [29] to generate mice bearing a post-thymic deletion of Themis combined with expression of the Vav1^R63W^ variant (KI-cKO mice). We first analyzed the numbers and percentages of thymocytes and T cell subsets in the thymus, spleen and lymph nodes from these mice. As previously described, cKO mice exhibited no major difference when compared to WT mice, except for the numbers of CD8 T cells, which were decreased in the spleen (Fig. S1). KI mice exhibited a slight defect of CD4^+^ thymocytes (Fig. S1C), fewer mature Tconv and CD8 T cells in the spleen (Fig. S1E) but no difference for those subsets in LN (Fig. S1D). Finally, the thymus of KI-cKO mice exhibited similar numbers of CD4^+^ thymocytes as KI mice, and similar CD8^+^ thymocytes as cKO mice (Fig. S1C). Similarly, the same numbers of KI-cKO Tconv in peripheral lymphoid organs were observed as in KI mice (Fig. S1D-E). However, KI-cKO mice had reduced numbers of CD8 T cells, in comparison to all other genotypes, suggesting a higher need for Themis and Vav1 for CD8 T cell maintenance (Fig. S1D-E). As previously reported, Vav1^R63W^ led to an increased frequency of Treg [29] in both genetic backgrounds (WT vs. cKO) (Fig. S1D–E). In contrast, the deletion of Themis had no effect on the frequency or the absolute numbers of Treg in the thymus (Fig. S1C), lymph nodes (Fig. S1D), and spleen (Fig. S1E), suggesting that Themis is dispensable for Treg homeostasis. Overall, even if the Vav1 variant and Themis deletion had some effect on T cell homeostasis at steady state, they did not lead to a strong lymphopenia as seen in germline KO mice for either Vav1 or Themis, enabling us to study without possible bias how the combination of those mutations could modulate peripheral CD4 T cell responses.

### Combined mutations in Themis and Vav1 genes reveal an epistatic interaction that modulate the susceptibility to CNS inflammation

**Fig. 1:**
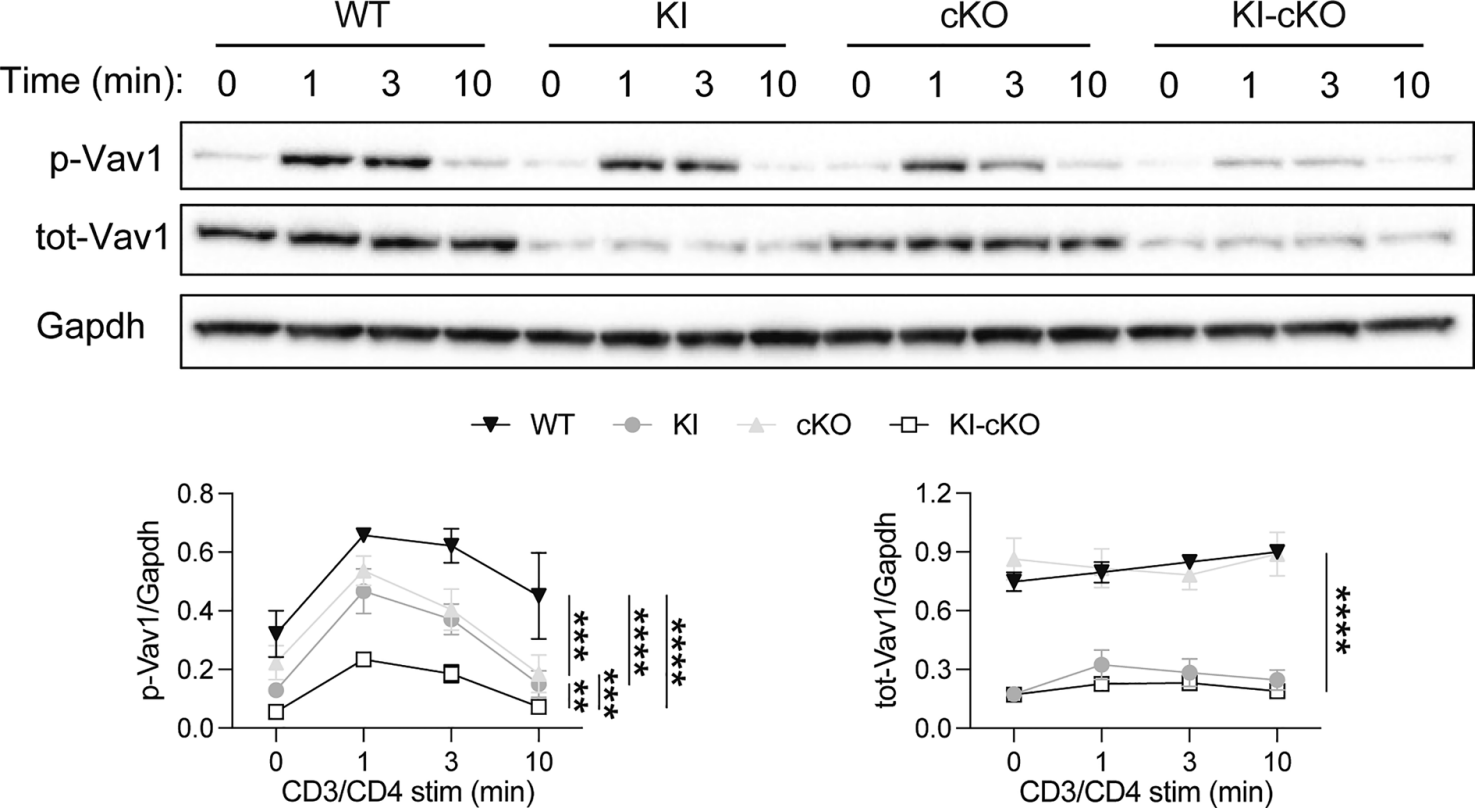
Themis positively controls Vav1 and Vav1^R63W^ phosphorylation in CD4 T cells. CD4 T cells were enriched from a pool of lymph nodes and spleen from naive mice of the indicated genotypes and stimulated for the indicated time with pre-formed anti-CD3/anti-CD4 antibodies complexes. Total and phosphorylated Vav1 were assessed by western blot and normalized to Gapdh. Results represent a pool of 4 independent experiments. Error bars represent SEM. Data were analyzed by two-way ANOVA (**p < 0.01, ***p < 0.001, ****p < 0.0001)

To investigate the impact of the combination of Themis deletion and Vav1^R63W^ mutation on Vav1 activity in CD4 T cells, we stimulated CD4 T cells from WT, cKO, KI and KI-CKO mice with anti-CD3 antibodies and compared the phosphorylation of Vav1 on its Tyrosine 174, which is reflective of its activity level [32]. As previously shown in thymocytes [7], the phosphorylation of Vav1 was reduced in the absence of Themis (Fig. 1). Confirming our previous observation, the Vav1^R63W^ variant induced a strong reduction in total protein amounts (Fig. 1), independently of Themis expression. We observed that the overall quantity of phosphorylated Vav1 was decreased in cKO CD4 T cells to a level similar to that in Vav1^R63W^ CD4 T cells (Fig. 1). Interestingly, the deletion of Themis in Vav1^R63W^ CD4 T cells decreased even more Vav1 phosphorylation, without impacting further the total protein quantity of Vav1, indicating an additive effect of the combined mutations on Vav1 activity (Fig. 1).

**Fig. 2:**
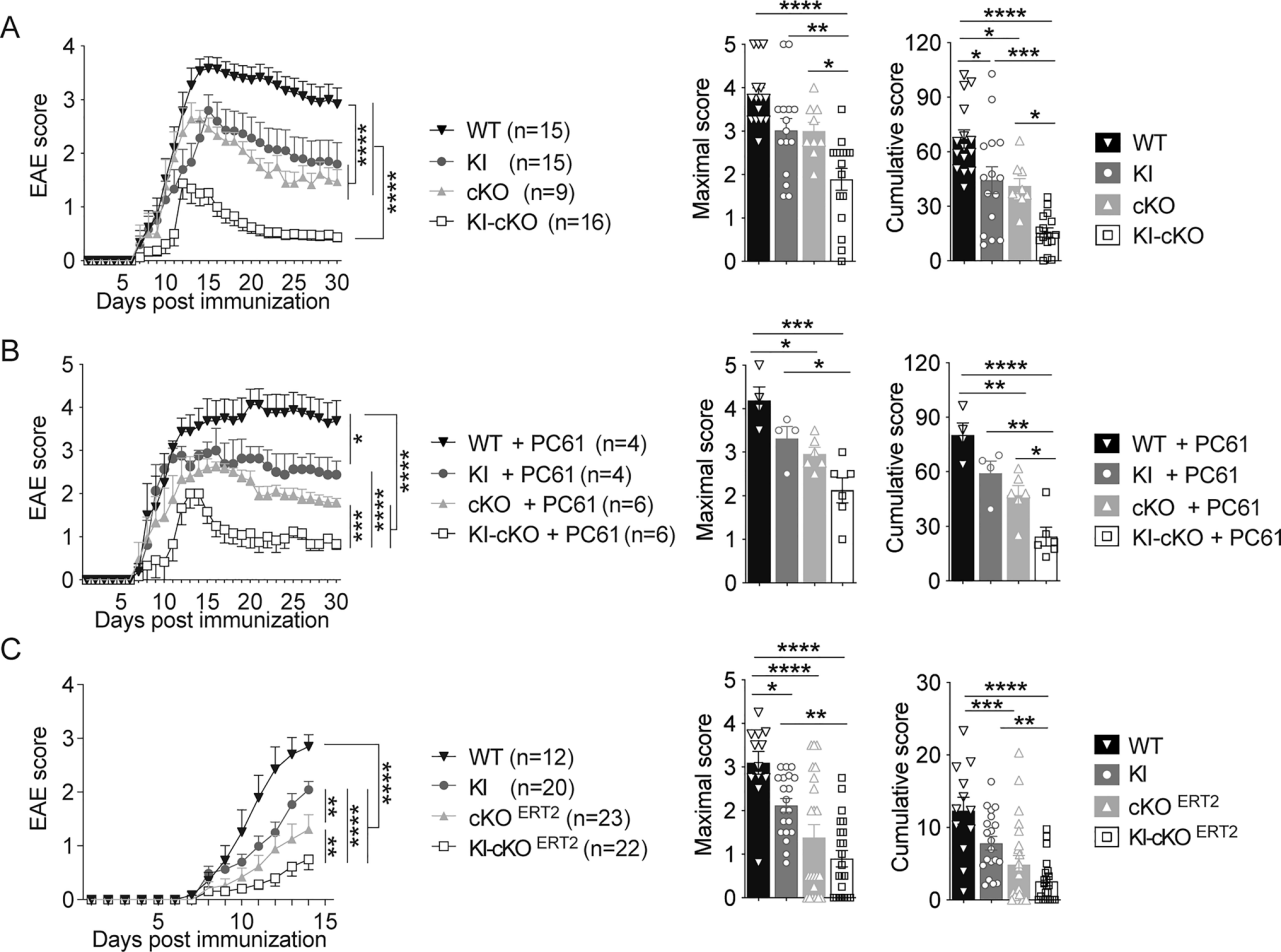
Combined mutations in Themis and Vav1 genes reveals an epistatic interaction that modulates the susceptibility to CNS inflammation **A.** WT, KI, cKO and KI-cKO mice were immunized with MOG_35–55_ peptide emulsified in CFA and clinical scores were evaluated daily. The data were expressed as mean of daily scores (left graphs), as mean of maximal scores (middle panels) or as mean of cumulative scores that represent the sum of daily clinical score of each mouse (right panel). The data are a pool of two independent experiments. **B** CD25^+^ Treg were depleted by treating mice from the four genotypes with PC61 antibody and immunized with MOG_35–55_ peptide 7 days later. **C** WT, KI, cKO^ERT2^ and KI-cKO^ERT2^ mice were treated with tamoxifen orally for 3 consecutive days and then immunized with MOG_35–55_ peptide 2 weeks later. The data are a pool of 3 independent experiments. Error bars represent SEM. Clinical curves differences were analyzed by two-way ANOVA. Maximal and cumulative scores histograms were analyzed by one-way ANOVA (*p < 0.05, **p < 0.01, ***p < 0.001, ****p < 0.0001)

We then assessed whether this gradual decrease of p-Vav1 in vitro could be correlated with a gradual in vivo phenotype, using a model of autoimmune neuroinflammation. To this end, we immunized WT, KI, cKO and KI-cKO mice with myelin oligodendrocyte glycoprotein peptide (MOG_35–55_) emulsified in CFA. As expected, WT mice developed a classical disease, characterized by a progressive ascendant paralysis, whereas KI and cKO mice developed a less severe EAE, as shown by the reduction of cumulative scores (Fig. 2A). This reduction of EAE severity was amplified in KI-cKO mice, as depicted by the delay in EAE onset and the less severe disease course compared to KI or cKO mice (Fig. 2A), suggesting a cooperative role of Themis and Vav1 during EAE development. Since Themis and Vav1 mutations could impact both Tconv and Treg compartments, we sought to determine if Tregs could be involved in the reduced EAE severity observed. We thus compared EAE development between the 4 genotypes after depletion of Treg with an anti-CD25 depleting mAb (Fig. S2). As expected, the disease was stronger without Treg but the difference in the disease severity was maintained between the four groups, demonstrating that CD25^+^ Treg are not responsible for the reduced EAE severity observed in KI, cKO and KI-cKO mice (Fig. 2B).

Although the CD2-driven Cre transgene begins to be expressed at a relatively late stage of T cell development [33], the effect of Themis on EAE development in cKO and KI-cKO mice could be the consequence of Themis deletion in CD8 T cells or of an altered selection of the TCR repertoire in the thymus. To evaluate these possibilities, we conditionally disrupted the *Themis* gene in peripheral CD4 T cells, by administrating tamoxifen to Themis^flox/flox^Vav1^WT^ and Themis^flox/flox^Vav1^R63W^ mice expressing the CD4-Cre^ERT2^-driven transgene [34] and compared the development of EAE in mice bearing single (KI and cKO^ERT2^) and combined (KI-cKO^ERT2^) mutations. We first verified that tamoxifen administration indeed induced a specific deletion of *Themis* gene in CD4 T cells, that was maintained all along the EAE protocol (Fig. S3). In agreement with the results obtained using the CD2-Cre driven transgenic model, we observed that the severity of EAE was decreased in cKO^ERT2^ and KI mice and was even decreased further in KI-CKO^ERT2^ mice when both Themis and Vav1 were mutated (Fig. 2C). In addition, by using adoptive transfer experiments of purified CD4 T cells from either WT or KI-cKO into RAG2-KO mice that were immunized with MOG_35–55_, we demonstrated the implication of CD4 T cells in the reduced EAE severity in KI-cKO mice (Fig. S3B). Together, these data show that the reduced EAE severity in KI-cKO mouse model is not the consequence of a defect of T cell development, neither of Themis deletion effect on CD8 T cells, but is rather caused by a defect in the CD4 T cell compartment.

### Double mutated KI-cKO Tconv exhibit a reduced capacity to infiltrate the CNS

**Fig. 3:**
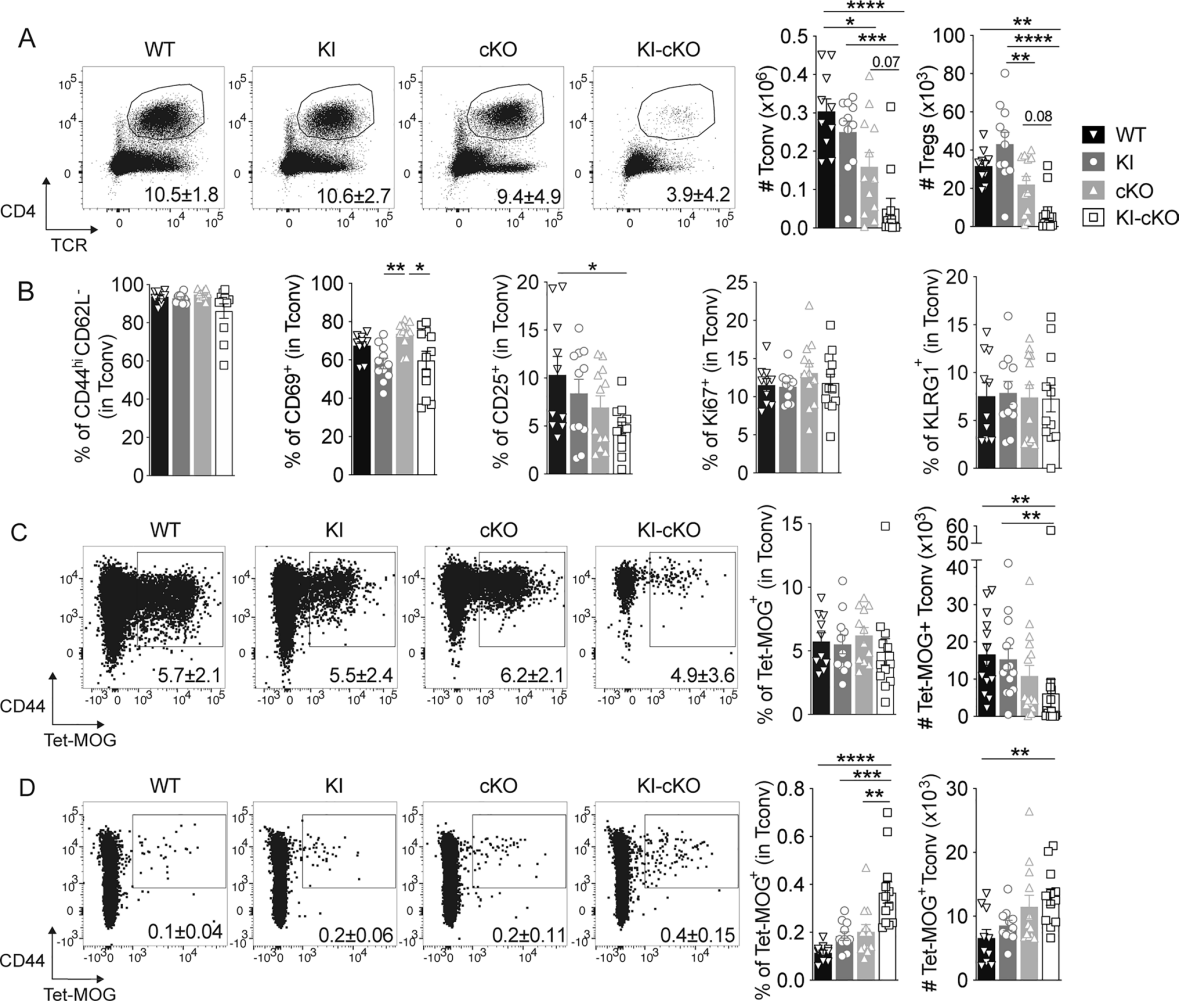
Double mutated KI-cKO CD4 T cells exhibit a defective capacity to infiltrate the CNS. Spinal cord (**A**–**C**) and draining lymph nodes (**D**) were collected from WT (n = 10), KI (n = 11), cKO (n = 12) and KI-cKO (n = 12) mice 14 days after MOG_35–55_ immunization. **A** Representative dot plots of TCR and CD4 expression in total cells from the four genotypes. Histograms represent the absolute numbers of conventional (Tconv) and regulatory (Treg) CD4 T cell subsets. **B** Expression of activation markers by Tconv. **C**, **D** Representative dot plots of MOG_35–55_ tetramer stanning of Tconv from the four genotypes. Histograms represent the percentages and absolute numbers of Tet- MOG^+^ Tconv in the spinal cord (**C**) or draining lymph nodes (**D**). Data are a pool of two independent experiments. Error bars represent SEM. Data were analyzed by one-way ANOVA (*p < 0.05, **p < 0.01, ***p < 0.001, ****p < 0.0001)

Activation and subsequent migration of autoreactive CD4 T cells into the CNS is a critical step in the pathogenesis of EAE and MS [35]. We analyzed immune cells isolated from the spinal cord and the draining lymph nodes 14 days after EAE induction (the peak of disease) (Figs. 3, S4). We observed a marked reduction in the numbers of infiltrating Tconv and Treg CD4 T cells in the spinal cord of KI-cKO mice as compared to that of WT, KI and cKO mice (Fig. 3A). The CD4 T cells that infiltrate the CNS in WT, KI, cKO and KI-cKO mice exhibited no major differences in activation status, as depicted by the expression of activation (CD44, CD62L, CD69, CD25), proliferation (KI67), or effector differentiation (KLRG1) markers (Fig. 3B). To analyze more specifically antigen-specific autoreactive T cells, we used MOG_35–55_-bearing tetramers to stain Tconv isolated from the CNS and dLN. We observed that the percentages of MOG-specific Tconv were similar in the spinal cord of the four groups, while the absolute numbers of MOG-specific Tconv were significantly decreased in the spinal cord of KI-cKO mice as compared to WT and KI mice (Fig. 3C), suggesting a general decrease in CD4 T cell infiltration, independently of their specificity. In agreement with this hypothesis, we observed a major reduction of multiple myeloid cell population, including polynuclear neutrophils and monocyte-derived cells, in the spinal cord of KI-cKO mice (Fig. S5), indicating an extensive reduction in all CNS-infiltrating immune cells. Interestingly, the reduced numbers of MOG-specific Tconv in the spinal cord at day 14 is associated with their accumulation in the draining lymph nodes (Fig. 3D) suggesting a failure fail to migrate efficiently into the CNS. Altogether, our data establish that the T cell-conditional deletion of Themis combined with the natural variant of Vav1 reduces EAE susceptibility by decreasing infiltration of encephalitogenic Tconv into the CNS, thereby likely contributing to a decreased CNS infiltration of all other cell types.

### MOG-specific Tconv from KI-cKO exhibit reduced effector functions during the priming in dLN

**Fig. 4:**
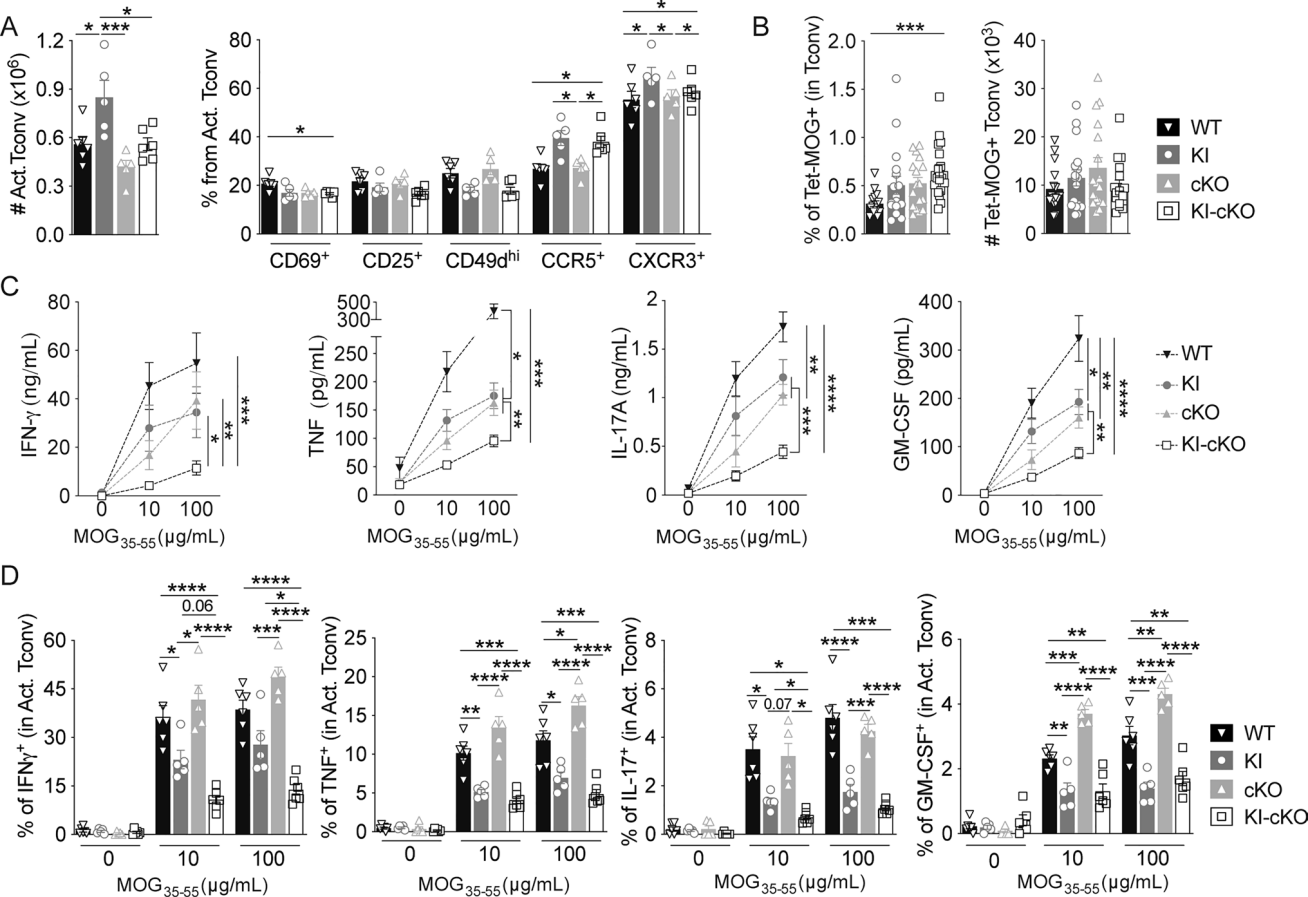
MOG-specific CD4 T cells from KI-cKO mice exhibit reduced effector functions during the priming in the draining lymph nodes. Draining lymph nodes from WT (n = 16), KI (n = 17), cKO (n = 17) and KI-cKO (n = 18) mice were analyzed 7 days after MOG_35–55_ immunization. **A** Absolute numbers of activated Tconv (CD44^+^CD62L^−^) and their expression of activation and migration markers. **B** Percentages and absolute numbers of Tet-MOG^+^ Tconv. **C**, **D** Cytokine production was analyzed in the supernatant (**C**) or intracellularly by ICS (**D**) after restimulation of dLN with increasing doses of MOG_35–55_ for 24 h. **C** Supernatants were analyzed for cytokine production by ELISA (IFN-γ) or by CBA. (**D**) Cytokine production was analyzed by FACS, gated on CD44^high^ Tconv. Data are representative of three independent experiments (**A**, **D**) or are the pool of three independent experiment (**B**) or two independent experiments (**C**). ELISA and CBA were analyzed by two-way ANOVA. Error bars represent SEM. A, B and D were analyzed by one-way ANOVA (*p < 0.05, **p < 0.01, ***p < 0.001, ****p < 0.0001)

To investigate the mechanisms whereby Themis and Vav1 modulate the ability of Tconv to infiltrate the CNS, we assessed their impact on the priming of MOG-specific Tconv prior to their migration into the CNS. dLN were collected on day 7 after MOG_35–55_ immunization and Tconv were analyzed for the expression of molecules involved in T cell activation and migration (Figs. 4, S6). Even though Tconv cell numbers were slightly reduced in KI-cKO mice as compared to WT, KI and cKO mice (Fig. S7A), the absolute numbers of antigen-experienced CD44^+^CD62L^–^ Tconv were similar between WT, KO and KI-cKO mice and slightly increased in KI mice (Fig. 4A). This was associated with an increased proportion of activated and memory Tconv in mice expressing Vav1^R63W^, independently of Themis deletion (Fig. S7B). The expression of activation (CD69, CD25) markers among this CD44^+^CD62L^–^ Tconv population did not reveal significant differences specific to KI-cKO mice (Fig. 4A). After in vitro stimulation with MOG_35–55_ for 48 h, we observed that Tconv from the four genotypes generated similar percentage of CTV^–^ cells, and similar absolute numbers of activated Tconv, suggesting equivalent proliferative and survival capacities (Fig. S7D–E). Of interest, KI-cKO Tconv also exhibited a similar expression profile of all chemokine receptors and integrins that we tested (CCR2, CCR5, CCR6, CXCR3, and CD49d) as compared to KI Tconv, suggesting that they can indeed respond efficiently to migratory signals (Figs. 4A, S7C). At this time point, we did not observe any increase in absolute numbers of MOG-specific Tconv in KI-cKO mice as compared to KI or cKO mice (Fig. 4B) suggesting that the accumulation of KI-cKO cells arises at later time points during disease development, possibly due to a migration defect into the CNS of the MOG-specific T cells from KI-cKO mice.

We next investigated the ability of WT and mutated Tconv to produce the inflammatory cytokines IFN-γ, TNF, IL-17, and GM-CSF, which are known to modulate the development of EAE. Following in vitro restimulation with MOG_35–55_ peptide, we analyzed cytokine secretion in the cell culture supernatants (Fig. 4C), which reflects cytokine production during the whole stimulation period. We measured significantly lower levels of IFN-γ, TNF, IL-17, and GM-CSF in the supernatant of KI-cKO MOG_35–55_-restimulated cells as compared to KI, cKO and WT cells, while single mutations triggered an intermediate reduction. In complement of this cytokine analysis, we performed intracytoplasmic staining (ICS) analysis to evaluate the relative contribution of CD4 and CD8 T cells. Following in vitro restimulation with MOG_35–55_ peptide, we observed by ICS a reduced production of all four cytokines by activated Tconv from KI mice compared to WT and cKO mice (Fig. 4D). Of those cytokines, both IFN-γ and IL-17 were significantly more reduced in KI-cKO mice, with a marked effect on IFN-γ suggesting a preferential cooperation between Vav1 and Themis in the control of IFN-γ secretion. In contrast, the production of these cytokines by CD8 T cells is marginal and there are no differences between the four genotypes (data not shown). The analysis of transcription factors, T-bet and RORγt revealed a significant reduction of both as percentage and/or as MFI in antigen-experienced Tconv of KI-cKO mice, 7 days post immunization, with a stronger decrease in T-bet (Fig. S6D). Together, our data reveal that the reduced pathogenicity of KI-cKO Tconv is associated with reduced production of IFN-γ, TNF, IL-17, and GM-CSF, with a more robust alteration of IFN-γ secretion, linked with a reduced expression of T-bet.

**Fig. 5:**
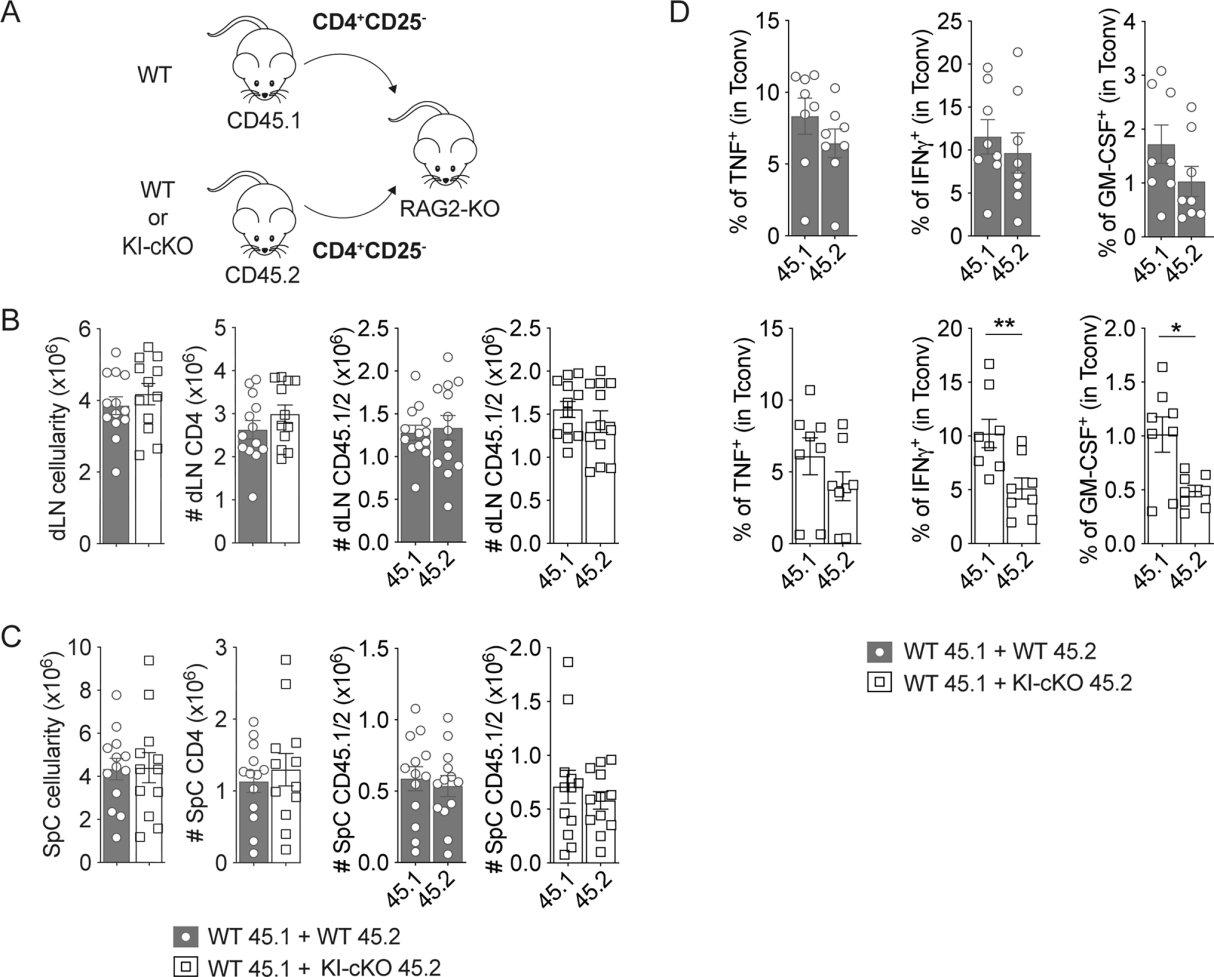
The deficit of CD4 T cell migration in KI-cKO mice is not due to lack of intrinsic migratory capacity. RAG2-KO mice were co-injected with two populations of TCR^+^CD4^+^CD25^−^ T cells (either WT CD45.1 + WT CD45.2 cells (n = 13) or WT CD45.1 + KI-cKO CD45.2 cells (n = 12)) and then immunized with MOG_35–55_. dLN and the spinal cord were analyzed 14 days after immunization. **A** Scheme of the experimental set-up. **B**, **C** Quantification of the absolute numbers of CD4 T cells in the draining lymph nodes (**B**) and the spinal cord (**C**). **D** Cytokine production by Tconv from the spinal cord analyzed by ICS following overnight restimulation with 100 μg/mL of MOG_35–55_. Data are pooled from 2 independents experiments (**B**, **C**), or are representative of 2 independents experiments (**D**). Error bars represent SEM. Data were analyzed by Mann- Whitney (* < 0.05, ** < 0.01)

Since pro-inflammatory cytokines play a key role in the disruption of the BBB and in the initiation of inflammatory cell infiltration into the CNS, we wondered whether the defect of T cell migration in KI-cKO mice could be reversed by the presence of WT Tconv. To test this hypothesis, we used WT CD25^−^ CD4 T cells (to remove most Treg) and KI-cKO CD25^−^ CD4 T cells bearing, respectively, CD45.1 and CD45.2 congenic markers, and we co-transferred them in RAG2-KO mice before immunization with MOG_35–55_ peptide (Fig. 5A). Fourteen days post immunization, KI-cKO Tconv expanded similarly to WT Tconv in the draining LN (Fig. 5B). In addition, we observed similar numbers of WT and KI-cKO Tconv in the spinal cord of RAG2-KO mice (Fig. 5C). These results demonstrate that KI-cKO Tconv can migrate as efficiently as WT Tconv towards the CNS and thus are not impaired in their intrinsic migratory capacity. Interestingly, we detected lower percentages of KI-cKO Tconv expressing IFN-γ and GM-CSF, compared to WT cells (Fig. 5D), suggesting that the defect in cytokine production of KI-cKO Tconv is cell-intrinsic. Taken together, these data demonstrate that the decreased susceptibility of KI-cKO mice to EAE is associated with a decreased ability of MOG-specific T cells to migrate from the periphery to the CNS as a result of an altered production of inflammatory cytokines.

## Discussion

In this study, we used mouse models bearing single and combined mutations in the TCR signaling proteins Themis and Vav1 to study a possible epistatic interaction of two proteins which have been previously associated with the susceptibility to Multiple Sclerosis (MS) and which are known physical interactors. Our results show that combined mutations on Vav1 and Themis induces a strong reduction of the severity of Experimental Autoimmune Encephalomyelitis (EAE), a mouse model of MS, contrasting with the moderate effect of each single mutation. Functional studies revealed that this effect resulted from the decreased production of pro-inflammatory cytokines (IFN-γ, IL-17, TNF and GM-CSF), together with a reduced T cell infiltration in the CNS. This was correlated with the amounts of activated Vav1 induced following TCR crosslinking, which were moderately decreased in the context of single mutations and more dramatically dampened when both Themis and Vav1 were mutated. Our data reveal an epistatic interaction between Themis and Vav1, which appears essential to allow the generation of optimal TCR signals required for CD4 T cell functions.

Our study also revealed that Themis deficiency associated with Vav1^R63W^ variant significantly reduced the ability of encephalitogenic Tconv to produce IFN-γ, IL-17, TNF, and GM-CSF. This is particularly relevant considering the key roles played by these cytokines in the pathophysiology of both EAE and multiple sclerosis [36–40]. One important effect of IFN-γ, TNF and IL-17 in the context of CNS inflammatory diseases is to enhance the permeability of the BBB [41–44] and to promote the recruitment of inflammatory myeloid cells into the CNS, together with activation of resident cells [39, 45–51]. Accordingly, we observed that the combined mutations of Themis and Vav1 led to a major reduction in the infiltration of MOG-specific T cells, but also of all other immune-cell subsets within the CNS. These includes monocyte-derived cells, which have been shown to produce matrix metalloproteinases necessary to disrupt the glia limitans, hence allowing cell entry into the CNS parenchyma [52]. Thus, our results suggest that the reduced ability of MOG-specific Tconv to secrete inflammatory cytokines in KI-cKO mice could be responsible for the reduced infiltration of immune cells in the CNS. Supporting this possibility, we showed that the defect in T cell migration of KI-cKO Tconv could be overcome by the presence of WT Tconv indicating that KI-cKO Tconv do not exhibit a general intrinsic incapacity to migrate towards the CNS. In agreement, KI-cKO Tconv also exhibited a similar expression profile of all chemokine receptors and integrins that we tested (CCR2, CCR5, CCR6, CXCR3, and CD49d) as compared to KI Tconv, suggesting that they can indeed respond efficiently to migratory signals. Interestingly, KI-cKO Tconv still produced lower levels of inflammatory cytokine IFN-γ and GM-CSF when transferred with WT Tconv, indicating that the defect of cytokine expression is intrinsic to effector KI-cKO Tconv. Together, our data reveal that the cooperative interaction between Themis and Vav1 enhances the production of inflammatory cytokines by effector Tconv, thereby indirectly contributing to facilitate their migration into the CNS.

The study of the modulatory effect of Themis on TCR signaling gave ambiguous results [4, 9, 13, 53]. It was first proposed that Themis could act as an attenuator of TCR signaling, by preventing the transmission of strong signaling responses induced by low affinity thymic self-antigen that would trigger inappropriate signals and lead to negative selection [9]. Contrasting with this interpretation, other studies showed that Themis enhances TCR signaling in thymocytes [7, 8] and cytokine-induced maintenance of peripheral CD8 T cells [14, 16], through its CABIT (Cystein-containing, All-Beta in Themis) modules that promote and stabilize the oxidation of the catalytic cysteines of SHP-1, thereby maintaining SHP-1 in an inactive form [8, 15, 16]. Our present study confirms that Themis is a positive regulator of the TCR signaling in mature CD4 T cells, by positively controlling Vav1 and Vav1^R63W^ phosphorylation. SHP-1 interacts with proteins of the TCR signaling machinery, including Vav1, and has been proposed to operate within negative feedback loops that contribute to negatively regulate TCR signaling upon TCR engagement [54, 55]. The interaction between Vav1 and SHP-1 is mediated by phospho-tyrosine residues on Vav1 that are directly recognized by the phosphatase domain of SHP-1 (PTP) [30], but it could also be mediated though SHP-1 SH2 domains. Themis interacts with SHP-1 through its CABIT domains which binds directly to SHP-1 PTP [8]. The only mechanism of interaction that has not yet been clearly characterized concerns Themis and Vav1, even if mass-spectrometry experiments have identified Vav1 and Themis as preferential binding partners in both thymocytes and CD4 T cells [7, 25]. It remains unclear whether Themis binds directly to Vav1, or indirectly through molecular intermediates. Since Themis does not contain known phospho-tyrosine binding domains and remains poorly phosphorylated in primary T cells, we favor the possibility of an indirect interaction. It is conceivable that SHP-1 would play this role, binding to Themis on one side (SHP-1’s PTP–Themis’s CABIT) and to phospho-Vav1 on the other (SHP-1’s SH2–Vav1’s p-Y). This would favor the positive control of Vav1 activation by Themis1 through SHP-1 inactivation.

Antigen recognition and T cell activation by the TCR is one of the most well-defined pathways of the immune system, having been dissected in mice and humans with loss of- function alleles in many of the critical components. It is, however, not known how inherited defects in TCR signaling translate into immunopathology in some instances, but not in others [20, 56]. Our study offers a possible explanation, by highlighting the importance to associate genetic variants observed in different signaling molecules belonging to the same hub, rather than focusing on individual molecules. Indeed, we showed that the combined defects on both Vav1 and Themis affects the magnitude of effector T cell responses, while the disruption of one single molecule has only a moderate effect. This genotype-dependent gradual decrease of EAE severity correlated with decreased quantity of phosphorylated Vav1 in CD4 T cells, demonstrating that Themis is required for optimal Vav1 activation to generate pathogenic Tconv responses in the CNS. We believe that this might be physiologically relevant, since our previous study showed that the Vav1 risk allele is associated with increased mRNA expression levels in PBMCs from healthy donors and multiple sclerosis patients, which is positively correlated to IFN-γ and TNF expression [57]. In addition, even though we studied a deletion of Themis and not a SNP, polymorphisms affecting its coding sequence or its mRNA expression level have been identified and were associated to a higher susceptibility to the development of pathologies [18, 58]. This indicates that genetic variants affecting Themis-mediated functional outcomes do exist in the human population. Thus, one might speculate that among individuals with low Vav1 expression, having in addition a defect in Themis or any other defects in this pathway could result in reduced inflammatory cytokines production by their Tconv. Since T cells play a crucial role in maintaining immune system homeostasis, the Themis/Vav1 signaling hub could thus represent a checkpoint for T cell functions and play an important role in controlling immune responses against self-antigens, cancer, and infectious diseases. Besides, Themis and Vav1 also play an important role in thymic development of T cells and their deficiency might lead to primary immunodeficiencies, as shown for Vav1 [59]. We showed that the combined effects of Themis and Vav1 mutations lead to a more severe immunodeficiency than the individual gene mutations alone, suggesting that epistatic interactions between these genes might contribute to stronger immunodeficiency phenotype in individuals affected on both genes.

### Supplementary Information

Below is the link to the electronic supplementary material.

Supplementary file1 (TIF 20329 KB)

Supplementary file2 (TIF 15519 KB)

Supplementary file3 (TIF 23402 KB)

Supplementary file4 (TIF 15218 KB)

Supplementary file5 (TIF 23309 KB)

Supplementary file6 (TIF 29898 KB)

Supplementary file7 (TIF 15145 KB)

Supplementary file8 (DOCX 22 KB)

## Data Availability

The data animal models and materials are available on request.

## Abbreviations

BBB: Blood–brain-barrier
BN: Brown Norway
CABIT: Cysteine containing all beta in Themis
CFA: Complete Freund’s Adjuvant
CNS: Central nervous system
CTV: Cell trace violet
DC: Dendritic cell
dLN: Draining lymph node
EAE: Experimental autoimmune encephalomyelitis
FACS: Fluorescent activated cell sorting
Foxp3: Forkhead box P3
GM-CSF: Granulocyte/macrophages colony stimulating Factor
Grb2: Growth factor receptor-bound protein 2
GWAS: Genome Wide Association Study
IFNγ: Interferon gamma
IL-17: Interleukin 17
LAT: Linker for activation of T cells
LEW: Lewis
MHC-I/II: Major histocompatibility complex class-I/class-II
MOG: Myelin oligodendrocyte glycoprotein
MS: Multiple Sclerosis
PTX: Pertussis Toxin
SHP-1: Protein tyrosine phosphatase, nonreceptor type 6
SNP: Single nucleotid polymorphism
SpC: Spinal cord
T-bet: T-box expressed in T cell
Tconv: Conventional CD4 T lymphocyte (Foxp3^−^)
TCR: T Cell Receptor
Themis: Thymocyte expressed molecule involved in selection
TNF: Tumor necrosis factor
Treg: Regulatory T cells (Foxp3^+^)
Vav1: Vav guanine nucleotide exchange factor 1
