# Supplementary figures and images for: Positive regulation of Vav1 by Themis controls CD4 T cell pathogenicity in a mouse model of central nervous system inflammation
Source: Cell Mol Life Sci. 2024 Apr 2;81(1):161. doi: 10.1007/s00018-024-05203-5 (PMC10987373; doi:10.1007/s00018-024-05203-5)

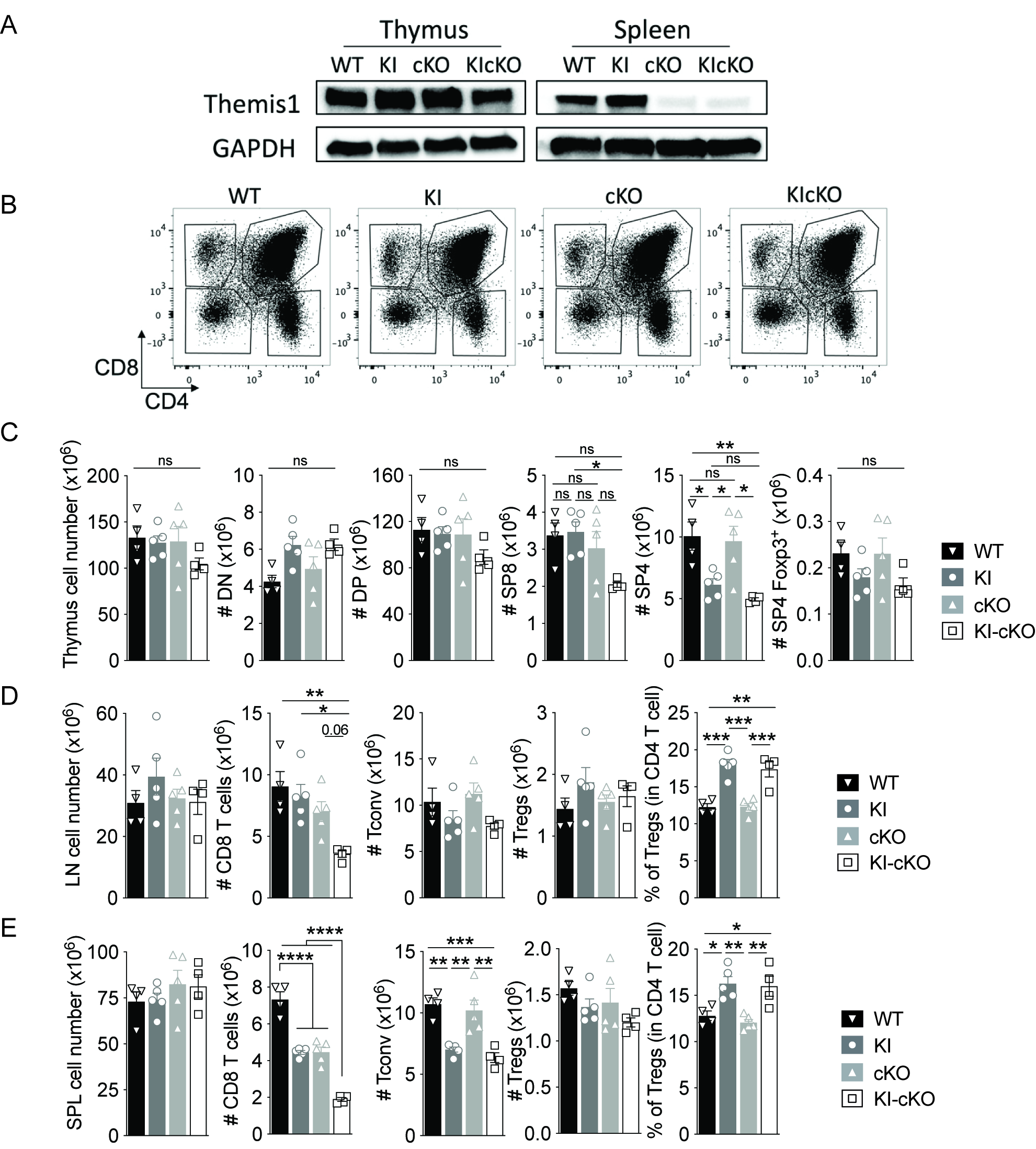

Supplement: Supplementary file 1 — Supplementary file1 (TIF 20329 KB) [file 18_2024_5203_MOESM1_ESM.tif]

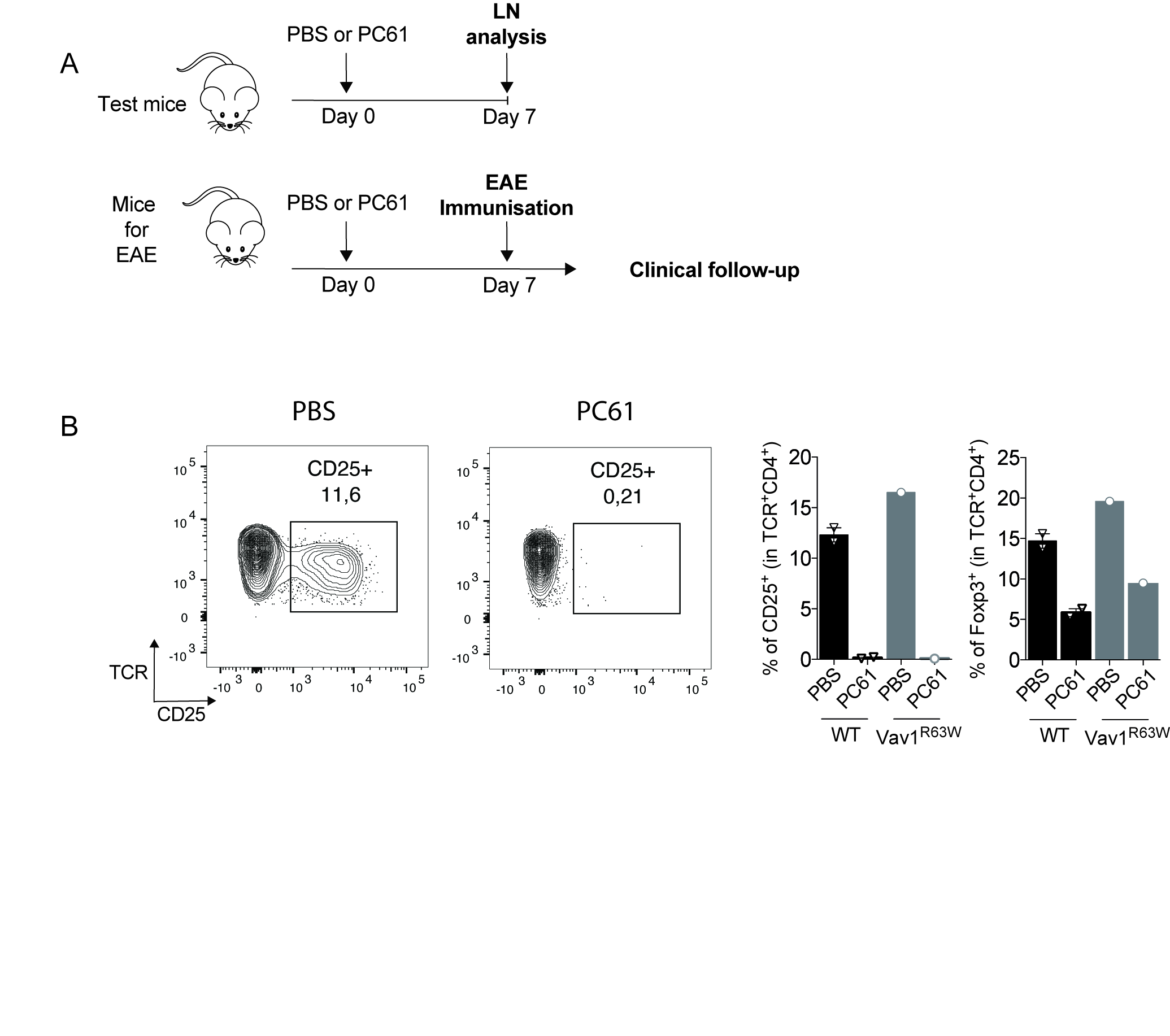

Supplement: Supplementary file 2 — Supplementary file2 (TIF 15519 KB) [file 18_2024_5203_MOESM2_ESM.tif]

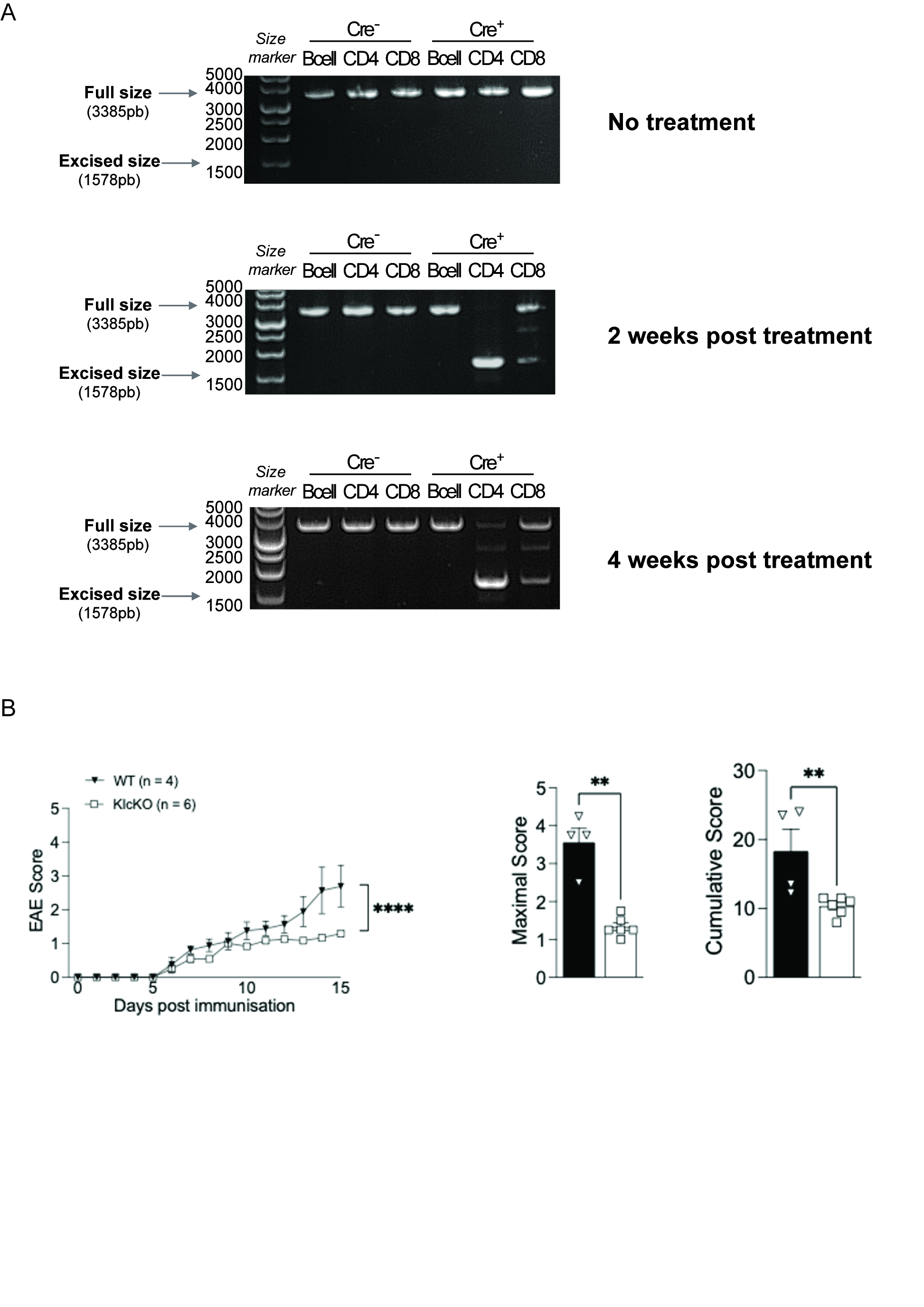

Supplement: Supplementary file 3 — Supplementary file3 (TIF 23402 KB) [file 18_2024_5203_MOESM3_ESM.tif]

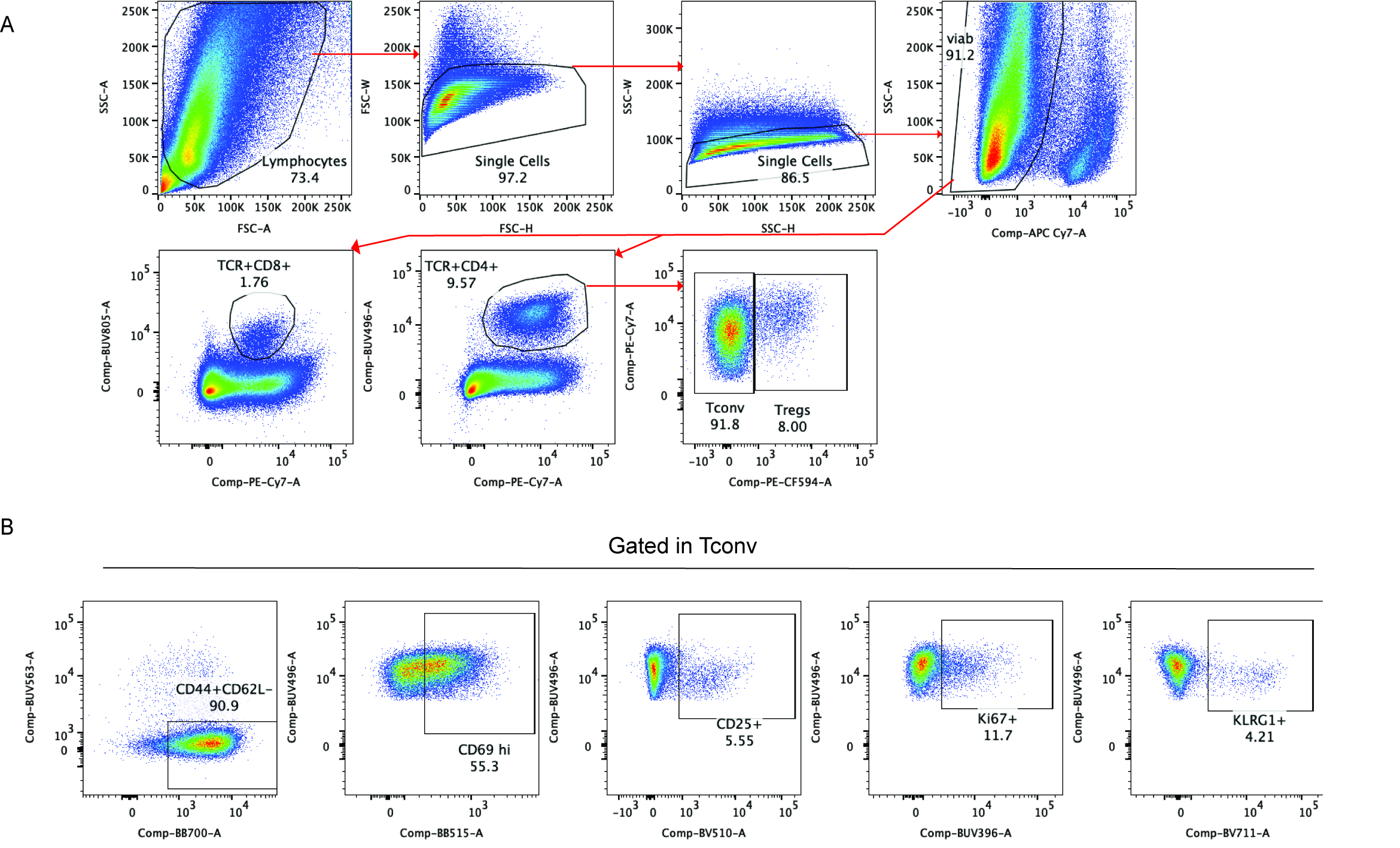

Supplement: Supplementary file 4 — Supplementary file4 (TIF 15218 KB) [file 18_2024_5203_MOESM4_ESM.tif]

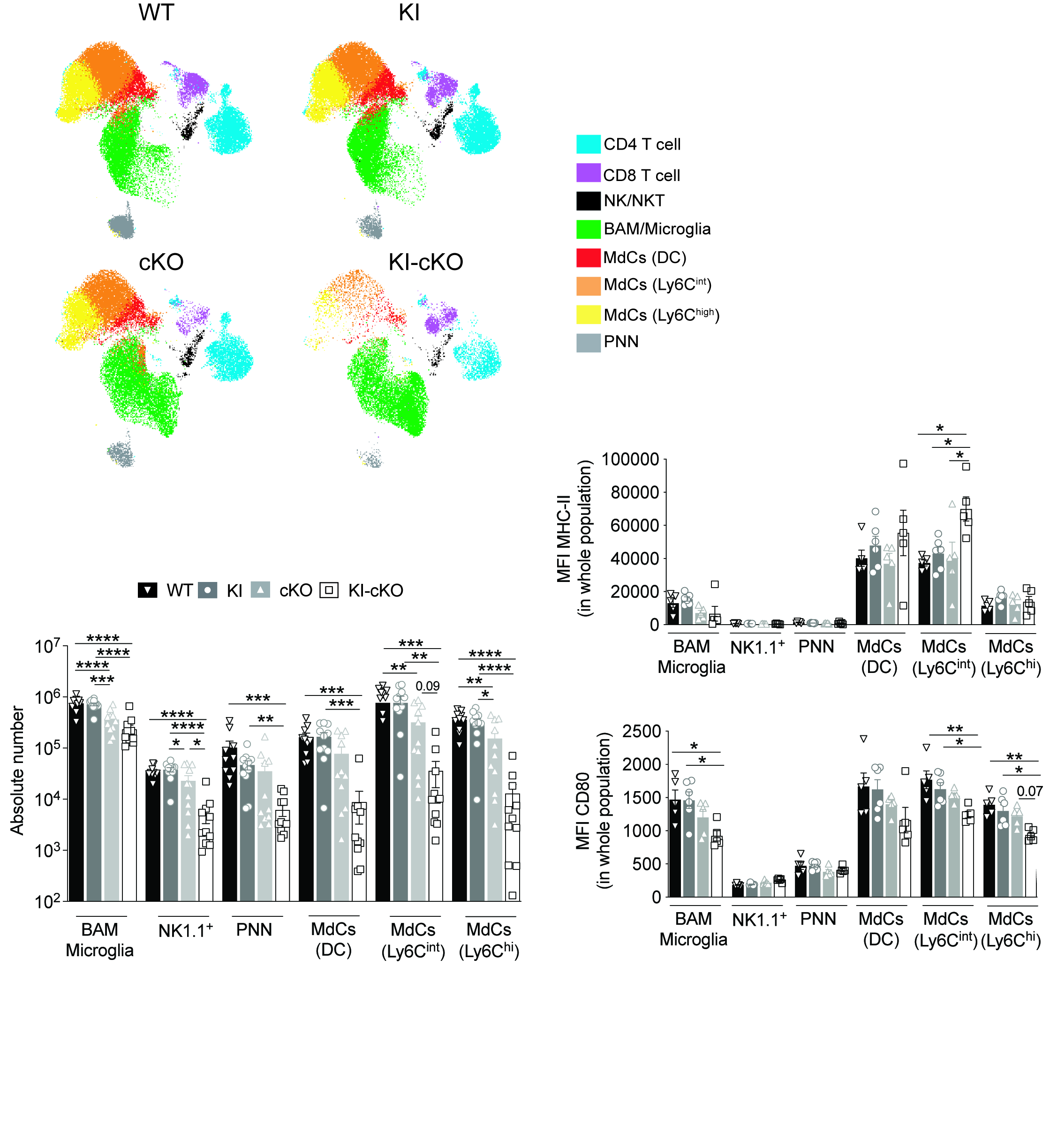

Supplement: Supplementary file 5 — Supplementary file5 (TIF 23309 KB) [file 18_2024_5203_MOESM5_ESM.tif]

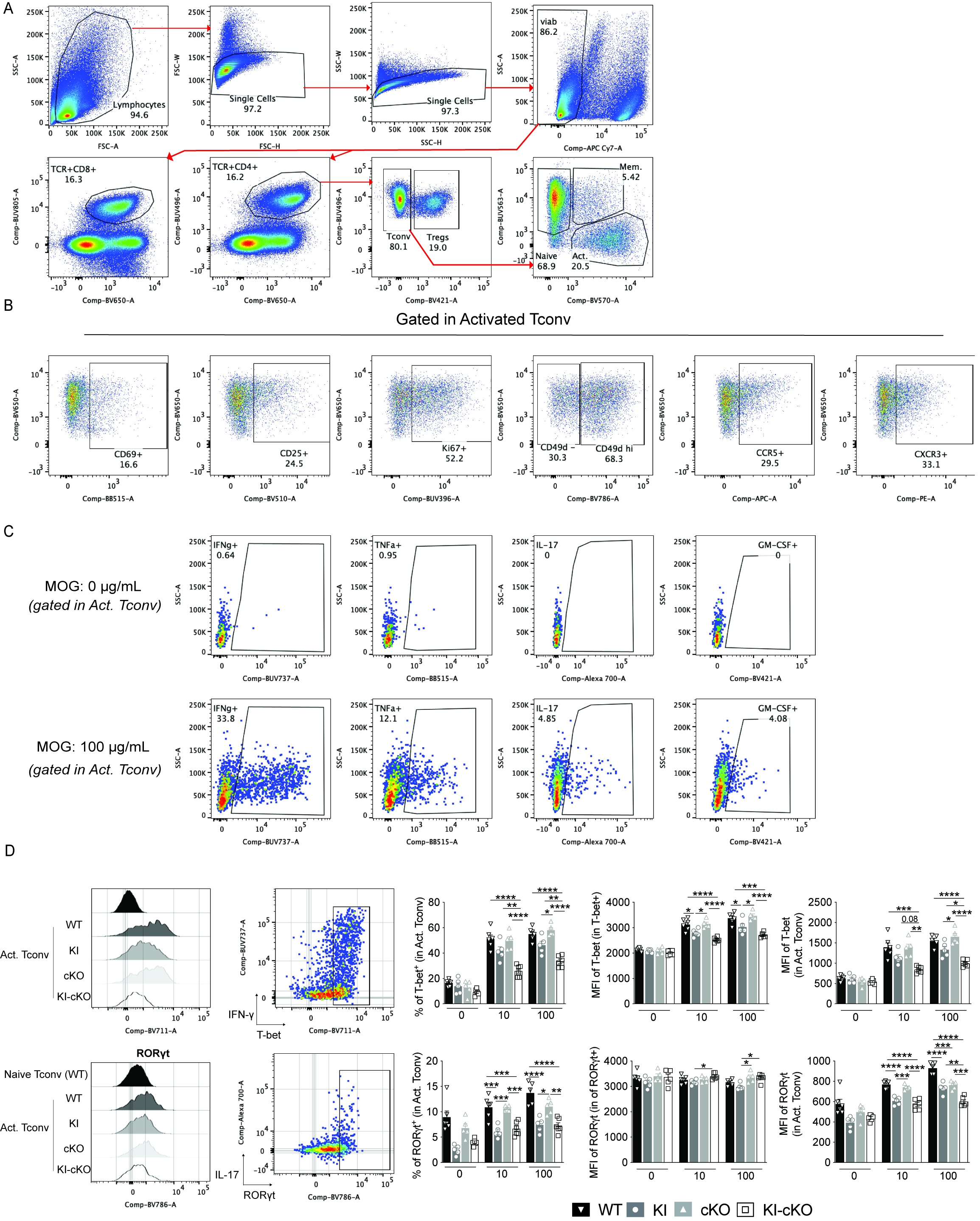

Supplement: Supplementary file 6 — Supplementary file6 (TIF 29898 KB) [file 18_2024_5203_MOESM6_ESM.tif]

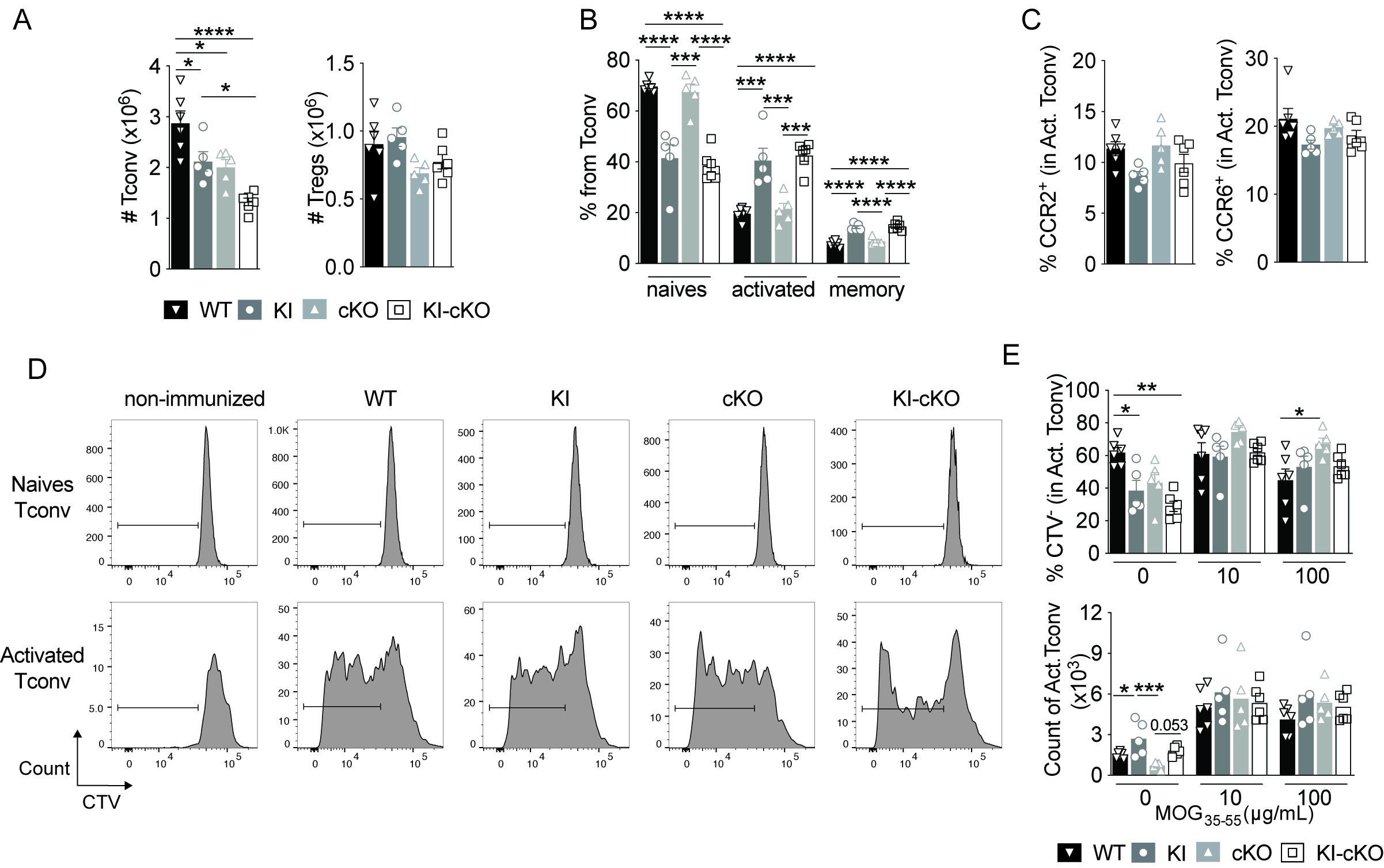

Supplement: Supplementary file 7 — Supplementary file7 (TIF 15145 KB) [file 18_2024_5203_MOESM7_ESM.tif]
