# Supplementary material for: Positive regulation of Vav1 by Themis controls CD4 T cell pathogenicity in a mouse model of central nervous system inflammation
Source: Cell Mol Life Sci. 2024 Apr 2;81(1):161. doi: 10.1007/s00018-024-05203-5 (PMC10987373; doi:10.1007/s00018-024-05203-5)
Supplement: Supplementary file 8 — Supplementary file8 (DOCX 22 KB) [file 18_2024_5203_MOESM8_ESM.docx]

**Fig. S1. Impact of combined mutations in Themis and Vav1 genes on T cell homeostasis**

Analysis of the thymus, spleen and lymph nodes of 6 weeks-old WT, KI, cKO and KI-cKO mice. (A) Total thymocytes (left) or splenocytes (right) were lysed and analyzed by western blot for expression of Themis1. (B) Representative dot plots of CD4 and CD8 expression gated on total thymocytes. (C-E) Graphs represent the absolute numbers and frequency of the indicated populations from the thymus (C), lymph nodes (D) and spleen (E). Error bars represent SEM. Data were analyzed by one-way ANOVA (*p<0.05, **p<0.01, ***p<0.001, ****p<0.0001).

**Fig. S2. PC61 treatment depletes CD25^+^ Treg from the dLN**

WT and KI mice were injected with PBS or PC61 antibody to test the depletion efficiency in the lymph nodes 7 days after injection. (A) Scheme of the experimental set-up of the depletion test and the depletion prior immunization. (B) Representative dot plots of TCR and CD25 expression within CD4 T cells of WT mice from the indicated group and quantification of CD25+ cells and Foxp3+ cells among CD4 T cells. Error bars represent SEM

**Fig. S3. Specific deletion of Themis in CD4 T cells following tamoxifen administration**

(A) Different immune populations were sorted from WT and cKOERT2 mice before (top) or after (middle and bottom) treatment with tamoxifen by oral gavage. DNA was then extracted from the indicated populations and subjected to PCR using primer pairs flanking the two loxP sites within Themis1 gene. The 3.4 Kpb PCR product corresponds to the WT unexcised Themis allele. The 1.6 Kpb PCR product corresponds to the Exon4-excised Themis allele. (B) Total CD4 T cells from WT (n=4) and KI-cKO (n=6) were injected i.v. in RAG2-KO mice which were immunized 7 days with MOG35-55 peptide emulsified in CFA and clinical scores were evaluated daily. The data were expressed as mean of daily scores (left graph), as mean of maximal scores (middle panel) or as mean of cumulative scores that represent the sum of daily clinical score of each mouse (right panel). Error bars represent SEM. Clinical curves differences was analyzed by two-way ANOVA. Maximal and cumulative scores histograms were analyzed by Mann-Whitney **p<0.01).

**Fig. S4 Gating strategy in the spinal cord**

Gating strategy used to analyze the data shown in Fig.3. All dot plots and histograms come from a WT mouse. (A) Doublet and dead cells were excluded. Shown is the gating strategy to identify CD8 T cells, total CD4 T cells, Tconv and Treg. (B) Analysis of activation markers among total Tconv, from last gate shown in A.

**Fig. S5 Myeloid cell infiltration in the CNS is reduced in KI-cKO mice**

Spinal cord was collected from WT, KI, cKO and KI-cKO mice 14 days after MOG35-55 immunization and stained to quantify the amount of resident microglia/BAM (border associated macrophages) and infiltrating myeloid cells. Each UMAP concatenates data from a pool of 5 mice. Microglia (Lin^-^ Ly6G^-^CD45^low^CD11b^low^CD44^low^), Polynuclear Neutrophils (Lin^-^Ly6G^+^), and different subpopulations of Monocyte-derived-cells (MdCs) (Lin^-^Ly6G^-^CD45^hi^CD11b^hi^CD44^hi^) were quantified and analyzed for their expression of MHC-II and CD80. Data are pool of two independent experiments (Absolute number) or representative of two experiments (MHC-II and CD80 MFI). Error bars represent SEM. Data were analyzed by one-way ANOVA (*p<0.05, **p<0.01, ***p<0.001, ****p<0.0001).

**Fig. S6 Gating strategy in the draining lymph nodes**

Gating strategy used to analyze the data shown in Fig.4. All dot plots and histograms come from a WT mouse. (A, B) show the ex vivo staining while (C, D) show respectively the cytokine staining or T-bet/RORγt staining post restimulation. (A) Doublet and dead cells were excluded. Gating strategy to identify CD8 T cells, total CD4 T cells, Tconv and their activation status. (B) Analysis of activation markers among activated Tconv (CD44^+^ CD62L^-^), from last gate in A. (C) Cytokine expression by activated Tconv originating from a MOG35-55 immunized WT mouse, in presence (bottom) or absence (top) of MOG_35-55_ peptide for restimulation. (D) Draining lymph nodes were harvested from WT, KI, cKO and KI-cKO mice 7 days after MOG35-55 immunization. Analysis of T-bet (Top panels) and RORγt (bottom panels) within antigen-experienced CD44^+^ CD62L^-^ Tconv by FACS following MOG35-55 restimulation of dLN cells for 48 h. Error bars represent SEM. Data were analyzed by one-way ANOVA (*p<0.05, **p<0.01, ***p<0.001, ****p<0.0001).

**Fig. S7. Characterization of Tconv activation during the priming phase**

Draining lymph nodes were analyzed from WT, KI, cKO and KI-cKO mice 7 days after MOG35-55 immunization. (A) Absolute numbers of the indicated cell populations. (B) Percentages of naive, activated and memory cells within Tconv, based on CD44 and CD62L expression. (C) Expression of chemokine receptors by activated Tconv. (D-E) Total dLN were stained with CTV for 10 min at 37°C prior to restimulation for 48 h with increasing doses of MOG35-55. (D) Representative dot plots of CTV dilution within naive (top) or activated (bottom) Tconv, 48 h after MOG35-55 restimulation. (E) Quantification of CTV^-^ cells within activated Tconv (top) and absolute numbers of activated Tconv recovered from each well at the end of the cell culture. Data are representative from one experiment. Error bars represent SEM. Data were analyzed by one-way ANOVA (*p<0.05, **p<0.01, ***p<0.001, ****p<0.0001).
